# Miss Lena Ashwell's Concert Parties

**Published:** 1915-08-28

**Authors:** 


### IN A GENERAL HOSPITAL IN FRANCE.

### Miss Lena Ashwell's Concert Parties.

One of the minor problems of hospital administration in the British Expeditionary Force, yet an important one in its way, is the amusement of the patients, upon whose hands time is apt to hang very heavy when once the acute stage of their illness is over. Especially at busy times, when frequently arriving convoys of new cases compel the surgeons to spend the major portion of each twenty-four hours in the operating theatres, hospital commandants and medical officers are apt to put off the arrangement of ward concerts from day to day, and then from week to week; and even in times of lesser strain it is a very easy matter to get "fed up," and to regard without enthusiasm or energy the prospect of a concert where each item has been contributed by the same performer three or four times on previous occasions of the same sort. Nevertheless, ward concerts of the amateur sort *are* got up not infrequently, and fresh "talent" *is* occasionally unearthed in the process; a "padre" accustomed to managing choir practices, and musically inclined, is a most valuable asset on such occasions.

Much as improvised concerts are appreciated by the patients, there is another kind which evokes greater enthusiasm, and is much more of an "event" in the hospitals, convalescent camps, and base depôts of all kinds in France. Need it be said that the reference is to the concert parties sent out under Miss Lena Ashwell's management and the auspices of the Y.M.C.A.? These parties of professional singers and players are dispatched from home about once a month, and make a round of the principal towns and ports used as bases for the Force, lasting about three weeks. This involves very hard work indeed for the performers, and a great tax on their energies and strength. But the writer has more than once been assured by them that the warmth and sincerity of the welcome which they receive wherever they go are ample compensation for this, and can well believe the statement. It would be, perhaps, an impertinence on his part to pay well-deserved compliments about the genuine merits of performance and performers; but he cannot refrain from testifying to the pleasure which the soldiers gain from their visits, and the real gratitude which they feel.

When the arrival of one of these concert-parties is expected at a general hospital, preparations are duly made for enabling as many patients as possible to attend. In summer weather the most sheltered outdoor space available is usually selected; at other seasons the largest ward is chosen and prepared as a concert-room. Patients unable to get up are transported in their beds by willing helpers and allotted the space nearest the "platform": sometimes a hundred or more beds full of wounded soldiers are grouped in rows in this manner. Further behind are chairs and forms for the patients who are able to get out of bed, with a corner reserved, in all probability, for the nursing and medical staffs. A piano has been produced from somewhere or other, and though it is not always of Queen's Hall quality, it is usually fairly in tune. On one occasion that the writer knows of the nearest piano-tuner was a blind Frenchman ten miles away: he was promptly sent for, lest one of Miss Ashwell's concert-parties should be incommoded by a piano whose intervals were not exactly those of the chromatic scale. A smattering of orderlies and of civilians (French, of course, mainly) completes the audience.

There are, as a rule, four singers—soprano, contralto, tenor, and baritone or bass commonly. One of the ladies, at least, is usually adept at ragtime and other popular melodies of the less classical kind; often a duet or a quartet is part of the programme. A funny man is, of course, almost indispensable, and is practically always forthcoming. Often he is a ventriloquist, with local colour imported into his patter—references to Tickler's jam are certain to raise a laugh. Sometimes the humorist is a teller of quaint stories, or can imitate all the noises of a farmyard or the approach of the different kinds of shell, or sings songs to his own accompaniment, in the Fragson or Margaret Cooper vein.

Then there is nearly always a violinist or a 'cellist, usually, but not invariably, of the fair sex. This instrument is always rapturously applauded and encored, proof that Tommy Atkins' taste in these matters is by no means so unsound as superior people might imagine. On one occasion the writer had charge of certain wards full of bedridden soldiers whom it was impossible to have transported to the concert; so he asked the violinist to visit these wards and play to the men there, which she very kindly did. On discovering that more than half the inmates of one ward were Scots, she demanded that they should choose a Scotch air for her to play. A fine old veteran thereupon produced from among his scanty possessions a tattered music-book, and requested that the "Bluebells of Scotland" should be played. The violinist played from the music, but at the conclusion of the air the patient remarked sadly, "That's no' the 'Bluebells o' Scotland.'" The lady protested that at least she had played the music as printed in his music-book, to which the Scot answered, "I'm no' saying that it's your fault, Miss; I'm thinking it's the music that's wrang." One must not omit to mention the very gifted accompanist, who also stage-manages the parties; his tact and popularity are equal to his piano-playing, which is saying a good deal.

As for the songs, "Tipperary" is by this time somewhat at a discount, and is seldom heard. "When Irish Eyes are Smiling" is nearly always included, though it is almost as hackneyed as the first-named; "Give Me Your Smile" is another pretty tune with stupid words, which has done yeoman service, and can do with a rest. "Vauxhall in the Morning" is a prime favourite; it is one of those songs to which only a really first-rate singer can do justice, and is, perhaps, that one of all the songs heard in France of which the writer cherishes the most pleasing memories. "Annie Laurie" is a song which the soldier never seems to tire of hearing; it always brings the house down, as also do "Sussex by the Sea," "We've come up from Zummerzet," "The Big Drum-Major," "When the Boys come Home," and other songs of the same type. The well-known chorus from the "Gondoliers" about the "regular Royal Queen" is often chosen by the quartet singers, and always goes down well, as also does "See what you want before you buy"; while "Madam, Will You Walk?" and "You're Here and I'm Here" are two of the favourite duets. On one not-to-be-forgotten occasion (within the writer's experience, that is to say, for it may have happened on other occasions) the party included Miss Ashwell herself, who contributed a fine recitation to the programme.

Early in July it was announced that funds were running short, and appeals were issued to the hospitals in France for contributions towards the cost of the parties. In one general hospital about twelve or thirteen pounds was immediately subscribed by the staff and by some officer-patients. The writer can affirm of his own knowledge that it would be a great pity if the parties were to cease coming out. At the same time, it is hardly fair to expect R.A.M.C. officers to provide further financial support for them, and it is "up to" the charitable in this country to see that Miss Lena Ashwell is supplied with funds for this valuable work that she is organising. If the Y.M.C.A. has no available funds, and those subscribed by Miss Ashwell's friends should run short, there might be a risk of these concerts being discontinued. Money for this purpose is just as usefully expended as if it were spent on splints and bandages, for diversion and amusement are valuable aids to recovery from bodily ills, whether they be fevers or bullet wounds. It would probably be true to say that these concert parties have actually saved lives; unquestionably they have brightened those of thousands of our soldiers just when they most needed diversion.

